# Remotely Self-Healable, Shapeable and pH-Sensitive Dual Cross-Linked Polysaccharide Hydrogels with Fast Response to Magnetic Field

**DOI:** 10.3390/nano11051271

**Published:** 2021-05-12

**Authors:** Andrey V. Shibaev, Maria E. Smirnova, Darya E. Kessel, Sergey A. Bedin, Irina V. Razumovskaya, Olga E. Philippova

**Affiliations:** 1 Physics Department, Lomonosov Moscow State University, 119991 Moscow, Russia; smirnova.me15@physics.msu.ru (M.E.S.); kes@polly.phys.msu.ru (D.E.K.); phil@polly.phys.msu.ru (O.E.P.); 2 Institute of Physics, Technology and Informational Systems, Moscow Pedagogical State University, 119435 Moscow, Russia; sa.bedin@mgpu.su (S.A.B.); irinarasum9@mail.ru (I.V.R.)

**Keywords:** polymer hydrogel, self-healing, magnetic gel, actuator

## Abstract

The development of actuators with remote control is important for the construction of devices for soft robotics. The present paper describes a responsive hydrogel of nontoxic, biocompatible, and biodegradable polymer carboxymethyl hydroxypropyl guar with dynamic covalent cross-links and embedded cobalt ferrite nanoparticles. The nanoparticles significantly enhance the mechanical properties of the gel, acting as additional multifunctional non-covalent linkages between the polymer chains. High magnetization of the cobalt ferrite nanoparticles provides to the gel a strong responsiveness to the magnetic field, even at rather small content of nanoparticles. It is demonstrated that labile cross-links in the polymer matrix impart to the hydrogel the ability of self-healing and reshaping as well as a fast response to the magnetic field. In addition, the gel shows pronounced pH sensitivity due to pH-cleavable cross-links. The possibility to use the multiresponsive gel as a magnetic-field-triggered actuator is demonstrated.

## 1. Introduction

Polymer hydrogels demonstrate highly responsive properties to various stimuli including temperature, pH-, magnetic field, and so on [1,2,3,4]. Of particular interest are magnetic-field-sensitive hydrogels (so-called ferrogels) [5,6,7,8,9,10], since they demonstrate rapid response to the stimulus and remote-control ability [11,12]. To provide magnetic-field responsiveness, magnetic particles like magnetite Fe_3_O_4_ [13], maghemite γ-Fe_2_O_3_, or cobalt ferrite CoFe_2_O_4_ [11,14,15] should be incorporated into the gel matrix. The resulting material combines the magnetic properties inherent to magnetic filler and the elastic properties inherent to hydrogels. When the magnetic field is applied, it induces the interaction between the particles, which leads to the change of the shape and of the elastic properties of the entire gel in a fast and reversible manner. It makes the magnetic-field responsive gels especially important for the development of remotely manipulated actuators for soft robotics [16], controlled valves [6], or plugs [8], and so on.

Magnetic hydrogels based on natural polymers are preferable over the other gels because of their enhanced sustainability, biocompatibility, biodegradation, and nontoxicity [12]. Biocompatible magnetic hydrogels are very promising as catheters for remotely controlled manipulation systems [17], microgrippers for excising cells and intravascular surgery [18], vehicles for magnetically guided drug delivery [19,20,21,22], and so forth. Biopolymer magnetic gels not only provide the possibility of biomedical applications, but also (which is even more important) significantly reduce the environmental pollution. Usually, such hydrogels are produced from various polysaccharides including sodium alginate [23,24], cellulose [22,25] and its derivatives [26], κ-carrageenan [27], chitosan [28,29] and its derivatives [30], agarose [31], starch [21], and some others.

Much less attention has been paid to guar gum polysaccharide. At the same time, it is cheap and produced in very large amounts from the seeds of Cyamopsis tetragonolobus [32], that is, from renewable biomass resources. Guar gum is a high-molecular weight non-ionic polysaccharide, which consists of α-(1→4)-linked-D-mannopyranose backbone with α-D-galactopyranosyl side groups attached by (1→6) linkages [32]. It easily forms gels upon the addition of various cross-linkers such as glutaraldehyde, phosphate, urea-formaldehyde, and borate [33]. Among them, borate is especially interesting, because it provides dynamic covalent cross-links with 1,2-cis-diol groups of polymer [34]. The enthalpy of formation of such bonds is rather low and equals 5–20 kT [35,36], which is much lower than the energy of “standard” covalent bonds [37]. As a consequence, borate cross-links are “labile” and reversibly break and recombine even at room temperature. Such labile cross-links provide faster response to external triggers as well as reshaping and self-healing ability that are highly requested in soft robotics.

Currently, there are only few papers describing guar gum magnetic gels. Most of them consider the combination of guar gum with synthetic polymers like polyacrylamide [38,39]. To the best of our knowledge, the magnetic guar gum gels without other polymer components were studied only in one paper [33], but these gels were formed only at elevated temperatures. This is useful for injection applications but significantly restricts the usage of these gels in many other areas, for instance, in the production of actuators. 

The aim of the present paper is to prepare at room temperature the magnetically actuated hydrogels with fast response to the magnetic field on the basis of a guar gum derivative—carboxymethyl hydroxypropyl guar (CMHPG). To provide fast response, we use dynamic covalent cross-links, which allow the polymer matrix to easily follow the displacement of magnetic particles under the action of a magnetic field. Additionally, a dynamic nature of the cross-links imparts to the gel reshaping and self-healing ability. In order to enhance the magnetic response, we use cobalt ferrite CoFe_2_O_4_ nanoparticles (NPs), which have higher magnetization than, for instance, magnetite NPs of the same size [40,41]; therefore, they are preferable for obtaining magnetic gels with strong response to magnetic field.

## 2. Materials and Methods

### 2.1. Materials 

**Figure 1:**
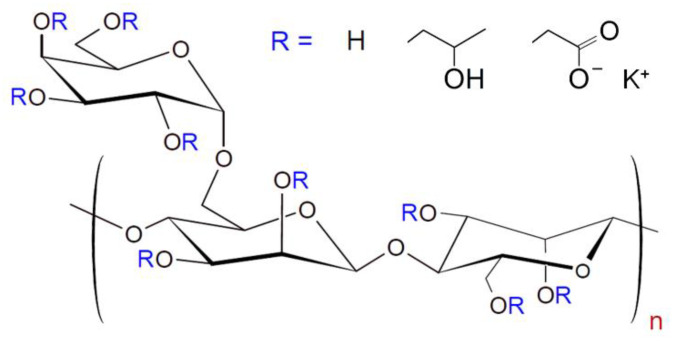
Chemical structure of carboxymethyl hydroxypropyl guar (CMHPG).

CMHPG POLYFLOS CH410P was provided by Lamberti (Gallarate, Italy). It is an anionic polyelectrolyte, derivative of a polysaccharide guar, bearing hydroxypropyl and carboxymethyl groups (Figure 1).

From NMR data, the degree of substitution of side α-D-galactopyranosyl units is equal to 0.8; the degrees of hydroxypropylation and carboxymethylation (numbers of hydroxypropyl and carboxymethyl groups per one monosaccharide residue) are 0.41 and 0.1, respectively [42]. According to viscometry, the molecular weight of CMHPG macromolecules is 1.6 × 10^6^ g/mol [42]. Hydroxypropyl guar (HPG) Jaguar HP105 from Solvay (Brussels, Belgium), boric acid (>99.9% purity) from ACROS (Geel, Belgium), potassium hydroxide (>98% purity) from Sigma Aldrich (St. Louis, MO, USA), and cobalt ferrite (CoFe_2_O_4_) NPs (99% trace metal basis) from Sigma Aldrich (St. Louis, MO, USA) were used as received. Solutions were prepared with deionized distilled water obtained on a Milli-Q device (Millipore, Burlington, MA, USA).

### 2.2. Polymer Purification

Aqueous CMHPG solution (0.5 wt%) was subjected to vacuum filtration through ceramic filters (ROBU GmbH, Hattert, Germany) with a pore diameter of 16–40 μm to remove water-insoluble impurities. Then, CMHPG was reprecipitated in ethanol (polymer solution:ethanol = 1:10 *v*/*v*). The precipitate was dissolved in water and lyophilized.

### 2.3. Preparation of the Samples

First, a desired number of NPs was suspended in KOH aqueous solution (pH 11). At this pH, CoFe_2_O_4_ NPs are negatively charged as a result of deprotonation of their surface -OH groups [43], and, therefore, they are electrostatically stabilized in the solution. In order to obtain a homogeneous dispersion, the solutions were ultrasonicated for 30 min with a Sonorex RK102H ultrasonic bath (Bandelin GmbH, Berlin, Germany) at 40 °C. Immediately after ultrasonication, dry polymer was added, and the solutions were mixed by a magnetic stirrer overnight. As a result, stable and homogeneous dispersions of NPs in polymer solutions (at polymer concentrations higher than 0.5 wt%) were obtained. Afterwards, a cross-linker (borax) solution (3.9 wt%, pH 11) was added, and the mixture was vigorously stirred with a spatula for 10–20 s and allowed to rest for a day, which resulted in the formation of cross-linked gels. Final pH of the samples was kept at 10.7 ± 0.1 by adding a small amount (1–3 μL) of 5M KOH solution when necessary.

### 2.4. Transmission Electron Microscopy (TEM)

**Figure 2:**
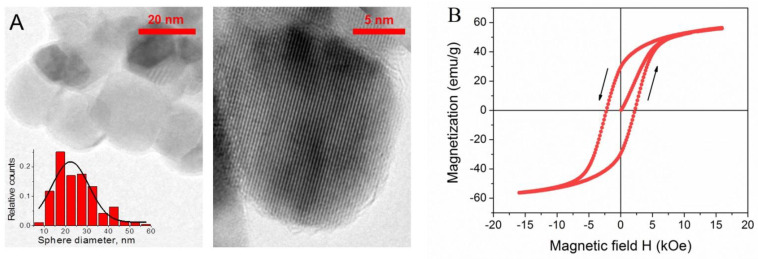
(**A**) TEM and HR-TEM images and histogram of the size distribution of CoFe_2_O_4_ NPs; (**B**) magnetic hysteresis curve of CoFe_2_O_4_ NPs at 297 K.

Transmission electron microscopy (TEM) and high resolution (HR-TEM) images were obtained using JEM 2100 F/Cs (Jeol, Tokyo, Japan) operated at 200 kV and equipped with UHR pole tip as well as spherical aberration corrector (CEOS, Heidelberg, Germany) and EEL spectrometer (Gatan, Munich, Germany). To prepare the TEM specimens, a drop (10 μL) of a pre-sonicated NPs aqueous solution was deposited onto a copper grid and air-dried under ambient conditions. Typical preparation of the TEM specimens is described elsewhere [44,45]. According to the TEM data, NPs are almost spherical and rather monodisperse, and their mean size equals to 20 nm (Figure 2A). HR-TEM images show that they are monocrystalline.

### 2.5. Magnetization Measurements

Magnetization curve of CoFe_2_O_4_ NPs was measured by a vibrating-sample magnetometer (VSM) 7407 (Lake Shore Cryotronics, Westerville, OH, USA)at room temperature in the range of magnetic field strengths ± 16 kOe. Dry NPs powder (5.4 mg) was sealed in a polyethylene container (4 × 4 × 0.5 mm) and laminated before measurements. NPs show hard ferrimagnetic behavior (Figure 2B) [46] and are characterized by a saturation magnetization M_s_ equal to 56.0 ± 0.5 emu/g, a rather large hysteresis loop with a high remanent magnetization M_r_ of 30 ± 0.5 emu/g, and a coercive force H_C_ of 2.2 ± 0.1 kOe. These magnetic properties are typical for single magnetic domain particles and are consistent with the data reported previously for CoFe_2_O_4_ NPs of similar size at room temperature [40,47].

### 2.6. Rheometry

Mechanical properties were investigated on a Physica MCR 301 rotational rheometer (Anton Paar, Graz, Austria). The experimental procedures are described in detail in [48,49]. Plate–plate geometry of the measuring cell was used. For the measurements in the absence of magnetic field, an upper plate had a diameter of 25 mm; and cylindrical gel samples with diameter of 25 mm and height of 3–4 mm were synthesized. The temperature, which was controlled using Peltier elements, was set to 20.00 ± 0.05 °C. To avoid evaporation of the solvent from the sample during measurements, a casing with Peltier elements was used. For the measurements in the presence of the magnetic field, a special plate–plate cell (MRD70/1T, Anton Paar, Graz, Austria) made from nonmagnetic material was utilized. The diameter of the upper measuring plate was 20 mm, and the gap was set at 0.7 mm. The magnetic field in the range of 0–1 T was generated in the measuring cell in the direction perpendicular to shear. The generated magnetic field was almost homogeneous at the sample length scale.

**Figure 3:**
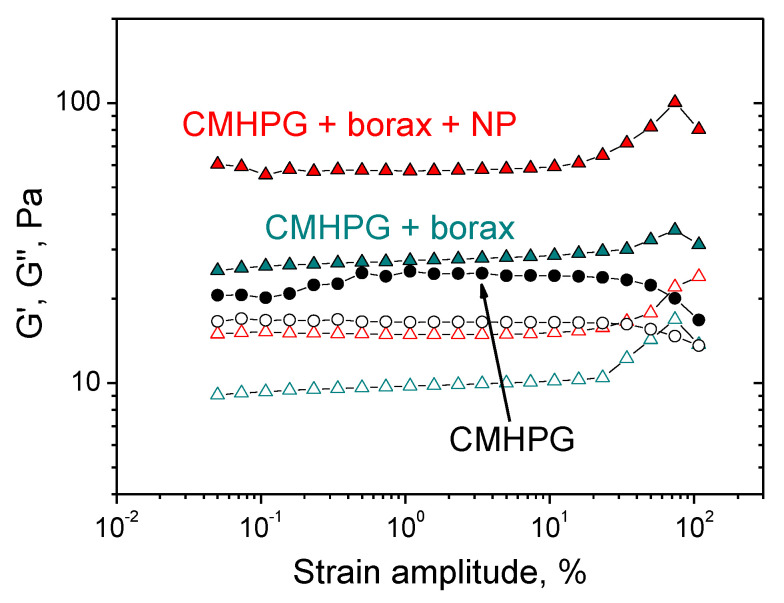
Amplitude dependences of storage G′ (filled symbols) and loss G″ (open symbols) moduli for aqueous solutions containing CMHPG (black symbols), CMHPG and borax (dark cyan symbols), CMHPG, borax, and CoFe_2_O_4_ NPs (red symbols). Concentrations: CMHPG—1 wt%; potassium borate—0.058 wt% (molar ratio (borate)/(monomer units) = 0.5); NPs—10 wt%. Temperature: 20 °C.

Experiments were carried out in the oscillation mode, in which the frequency dependences of the storage modulus G′ and the loss modulus G’’ were measured in the range of external impact frequency ω = 0.04–50 s^–1^. All measurements were performed in the linear viscoelasticity mode (at a strain amplitude of γ = 3–5%), in which the storage and loss moduli were amplitude independent. Linear viscoelastic range was determined by amplitude sweep measurements for CMHPG solution without borax and NPs, as well as for gels with borax, and with both borax and NPs (Figure 3).

### 2.7. Actuation Force Measurements

Force generated by the gels in the magnetic field was measured by a custom-made experimental setup equipped with a digital force meter 53002 (Megeon, Moscow, Russia) with measurement limits 0–2 H and accuracy 1 mH. A magnetic field (0–0.26 Tl) was generated by a permanent NdFeB magnet. During measurements, the sample and the magnet were moved away for at least 15 cm from the force meter, and the sample was connected to it by a non-magnetic spacer in order to avoid the influence of magnetic field on the force measurements.

## 3. Results and Discussion

In this article, we investigate hydrogels of CMHPG cross-linked by borate ions (borax) with added 20-nm magnetic CoFe_2_O_4_ nanoparticles. The concentration of CMHPG is fixed at 1 wt%, which corresponds to the semi-dilute regime [42]. In this regime, borate can cross-link different CMHPG macromolecules into a network due to the formation of dynamic covalent bonds with hydroxypropyl groups of the polymer [50]. Such cross-links are formed only at high pH > 9.2, e.g., above the pKa of boric acid, when it is transformed to borate [51]. Taking this into account, in the present work, the gels were prepared at pH 10.7. The concentration of NPs was varied in the range of 0–10 wt% (0–1.9 vol%).

### 3.1. Effect of NP Content

**Figure 4:**
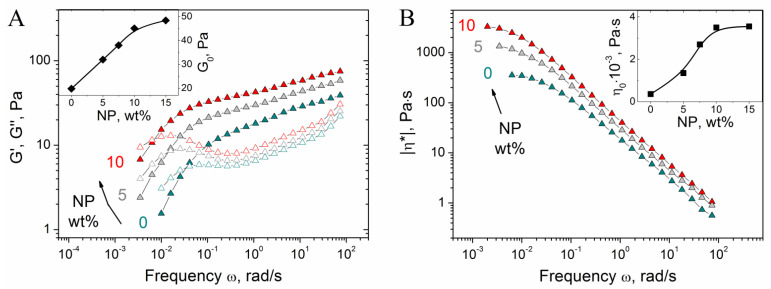
Frequency dependences of storage G′ (filled symbols) and loss G″ (open symbols) moduli (**A**) and modulus of complex viscosity |η*| (**B**) for hydrogels containing 1 wt% CMHPG, 0.058 wt% borax (molar ratio (borate)/(monomer units) = 0.5) and various concentrations of CoFe_2_O_4_ NPs indicated in the figure. Temperature: 20 °C. Inlets show the dependences of G_0_ (storage modulus at ω = 1.7 rad/s) and zero-shear viscosity η_0_ on NPs concentration.

First, we investigate the effect of NPs concentration on the properties of CMHPG/borate gels. In the absence of NPs, the gels show pronounced viscoelastic behavior (Figure 4A), which confirms the formation of a cross-linked polymer network: a cross-over point between G′(ω) and G″(ω) is observed due to the dynamic nature of borate cross-links, as a result of which the gels can flow. Such a behavior is typical for hydroxypropyl guar (HPG) gels cross-linked by borate [52].

Added NPs induce the increase of the storage modulus G’ (Figure 4A), suggesting that NPs form cross-links between CMHPG chains in addition to borate cross-links. At the same time, the gels retain viscoelastic behavior, meaning that the cross-links formed by NPs are reversible, similar to the borate cross-links. Addition of NPs also results in the shift of the cross-over point between G′(ω) and G″(ω) to the left, e.g., to the increase of the relaxation time (Figure 4A). This is explained by the fact that the movement of polymer chains is slowed down by their interaction with NPs, leading to the retardation of stress relaxation. Both these processes, formation of additional cross-links and slowing down of stress relaxation, contribute to the increase of viscosity (Figure 4B). As seen from the inlet in Figure 4B, zero-shear viscosity η_0_ (calculated from the low-frequency plateau value of the modulus of complex viscosity |η*|, which coincides with η_0_ for borate-crosslinked fluids [53,54]) increases with the rise of NP content. As mentioned above, this is related to the diffusion of polymer chains, which is slowed down by their interaction with NPs.

**Figure 5:**
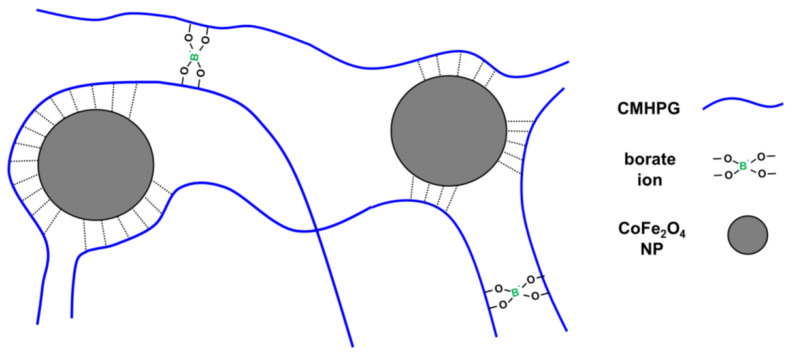
Scheme of formation of a CMHPG hydrogel dual-crosslinked by borate ions and CoFe_2_O_4_ magnetic NPs.

**Figure 6:**
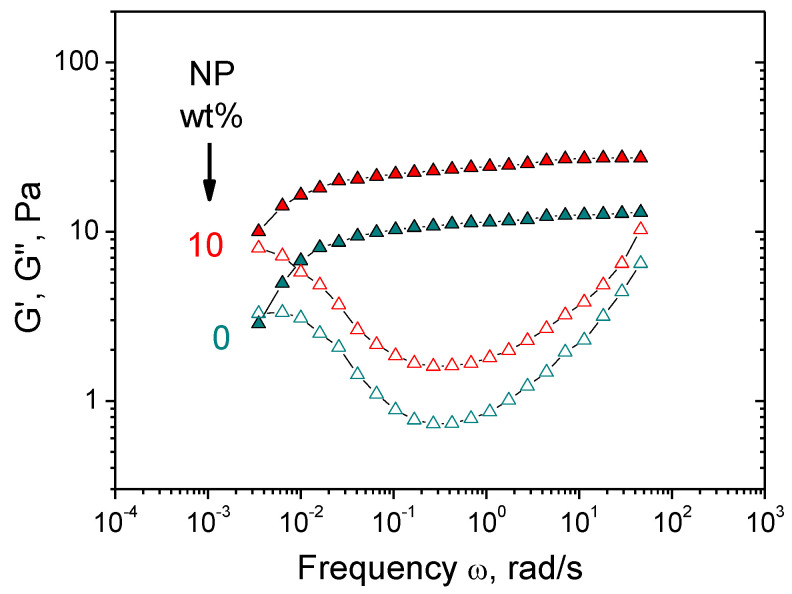
Frequency dependences of storage G′ (filled symbols) and loss G″ (open symbols) moduli for hydrogels containing 1 wt% HPG, 0.058 wt% borax (molar ratio (borate)/(monomer units) = 0.5) and various concentrations of CoFe_2_O_4_ NPs indicated in the figure. Temperature: 20 °C.

Therefore, in the presence of NPs, dual cross-linked gels are formed, in which borate ions and NPs serve as two types of dynamic cross-links (Figure 5). Cross-links by NPs are formed due to non-covalent interactions of macromolecules with their surface. CMHPG chains contain two types of functional groups—carboxylic and hydroxylic—which may be involved in the interaction with NPs. In order to elucidate which groups are responsible for cross-linking of polymer by NPs, a similar polymer—hydroxypropyl guar (HPG)—was taken, which bears only hydroxyl groups. As seen from Figure 6, NPs induce similar rheology enhancement of HPG solutions as for CMHPG. Therefore, it can be assumed that hydroxylic groups mainly participate in the polymer–NP interactions. The fact that carboxylic groups do not play a role in the binding of polymer to NPs may be explained by the fact that, at high pH 10.7, all carboxylic groups are deprotonated [42]. At the same time, cobalt ferrite has an isoelectric point at pH ~ 6.5; therefore, at pH 10.7, the NPs are also strongly negatively charged [55]. Thus, electrostatic repulsion exists between –COO^-^ polymer groups and NPs. Consequently, one can assume that the formation of cross-links by NPs is due to the interaction of hydroxylic groups of polymer with their surface. Literature data suggest that this interaction may be due to the formation of hydrogen bonds. Previously, guar absorption was observed on alumina, titania, and some other mineral-bearing surface –OH groups [56]. The formation of hydrogen bonds was observed in the case of HPG interacting with TiO_2_ NPs even at high pH ~ 10, when the NPs are negatively charged [57]. Though individual bonds between polymer and NP are weak, the resulting cross-links are rather strong and contribute to the storage modulus G′, since many groups can interact with one NP with the formation of a “multipoint” cross-link. 

The formation of cross-links by NPs is possible because of their rather small size (20 nm). Indeed, the degree of polymerization of CMHPG is ca. 2000 (assuming that one monomer unit is constituted by two monosaccharide residues in the main chain, with some side galactose units attached). Taking into account that the projection of the repeat saccharide unit on the main chain is ~0.52 nm [58], the contour length of a CMHPG macromolecule equals to ca. 2 μm. Water is a good solvent for CMHPG [59]; therefore, macromolecules are in expanded coil conformation. Their end-to-end distance R can be estimated by using the equation [60]

(1) $$R=l_{k}\;\times \;{n_{k}}^{3/5}=316\;\mathrm{nm}$$

where *l_k_* = 20 nm is the Kuhn segment and *n_k_* = *L*/*l_k_* = 100 is the number of Kuhn segments in the chain. For the Kuhn segment, an intrinsic value of 20 nm for guar was taken [61]. If polyelectrolyte effect, resulting from the presence of carboxymethyl groups, is taken into account, this would increase the length of Kuhn segment, and, correspondingly, the value of R. At 10 wt% of NPs, the mean distance between their surfaces is ca. 40 nm. Therefore, the size of the polymer coils is much larger than the distance between NPs. Consequently, one polymer chain can interact with several NPsand “connect” them together, which results in the increase of mechanical properties. Previously, in the literature, it was mentioned that small size of non-magnetic TiO_2_ particles is crucial for thickening of guar solutions [57].

Moreover, NPs are effective for the enhancement of viscoelastic properties of the gels, since one NP can possibly connect multiple macromolecules and serve as a “multifunctional” cross-link, contrary to one borate ion, which can link no more than two macromolecules. Indeed, the hydrodynamic radius of the polymer coil is [58]

(2) $$R_{h}{=(3\pi /128)}^{1/2}\;\times \;R\;\approx \;85\;\mathrm{nm}$$

The volume around one NP with a radius r = 10 nm, which can be occupied by macromolecules having contact with the NP surface, can be estimated as [62]

(3) $${V=4/3\pi \;[(2\mathrm{Rh}+r)}^{3}{\;\text{–}\;r}^{3}]$$

The number of macromolecules attached to one NP may be approximately calculated (assuming that polymer coils near the surface of NP do not overlap) as n_a_ = V/V_1_ ≈ 9, where V_1_ = 4/3πR_h_^3^ is the hydrodynamic volume of a single polymer coil.

Therefore, the use of rather small NPs as compared to the size of polymer coils allows obtaining dual physically cross-linked gels of CMHPG, where “single” cross-links are formed by borate ions, and “multiple” cross-links are represented by NPs. Addition of more NPs results in the enhancement of the mechanical properties of the gels. 

### 3.2. Effect of Borate Content

**Figure 7:**
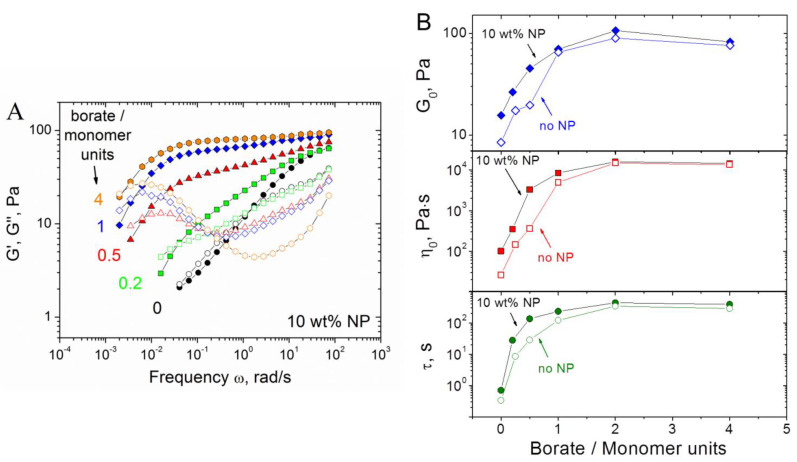
(**A**) Frequency dependences of storage G′ (filled symbols) and loss G″ (open symbols) moduli for hydrogels containing 1 wt% CMHPG, various concentrations of borate (indicated in the figure) and 10 wt% CoFe_2_O_4_ NPs. (**B**) Dependences of G_0_ (storage modulus at ω = 1.7 rad/s), η_0_ (zero-shear viscosity), and relaxation time (τ) on the molar ratio of borate to CMHPG monomer units in the absence (open symbols) and in the presence of 10 wt% CoFe_2_O_4_ NPs (filled symbols). Temperature: 20 °C.

In order to find the optimal conditions for the formation of CMHPG/borate/NP gels, the effect of borate concentration on the mechanical properties was studied. As seen from Figure 7A, the increase of borate concentration induces a significant enhancement of viscoelastic properties. Indeed, in the absence of borax, only very weak viscoelastic properties are seen, which are due to cross-links formed by NPs and some entanglements between polymer chains. Upon addition of borate, a high-frequency plateau at G′(ω) dependence appears and widens, whereas the cross-over point between G′(ω) and G″(ω) moves to the left, meaning the increase of the relaxation time. This shows that, in addition to NP cross-links, borate cross-links are formed, and their amount rises with increasing borate concentration, contributing to the formation of a more tightly cross-linked network.

From Figure 7B, it is seen that in a wide range of borate concentrations (molar ratios of borate to monomer units from 0 to ca. 2), the rheological characteristics (high-frequency storage modulus G_0_, zero-shear viscosity η_0_, and relaxation time τ) of the gels grow with increasing borate content. Additionally, these characteristics are larger in the presence of NPs than without them. It indicates that NPs form cross-links between polymer chains. 

At very high borate concentrations (molar ratios of borate to monomer units higher than 2), the rheological properties level off, which suggests that all accessible 1,2-cis-diol groups are involved in the interaction with borax (with the formation of both di-diol cross-links between one borate ion and two polymer chains, as well as mono-diol complexes between one borate ion and one macromolecule). Note that at these conditions, the rheological properties with and without NPs almost coincide. This may indicate that borate ions compete with NPs for 1,2-cis-diol groups of polymer; therefore, at the excess of borate, NPs cannot form cross-links. These results count in favor of our suggestion that hydroxyl groups of CMHPG are involved in the interaction with NPs.

Thus, the gels dually cross-linked by NPs and borax are formed in a wide range of borate concentrations, where the total number of accessible hydroxyl groups is rather large. 

### 3.3. Effect of pH

**Figure 8:**
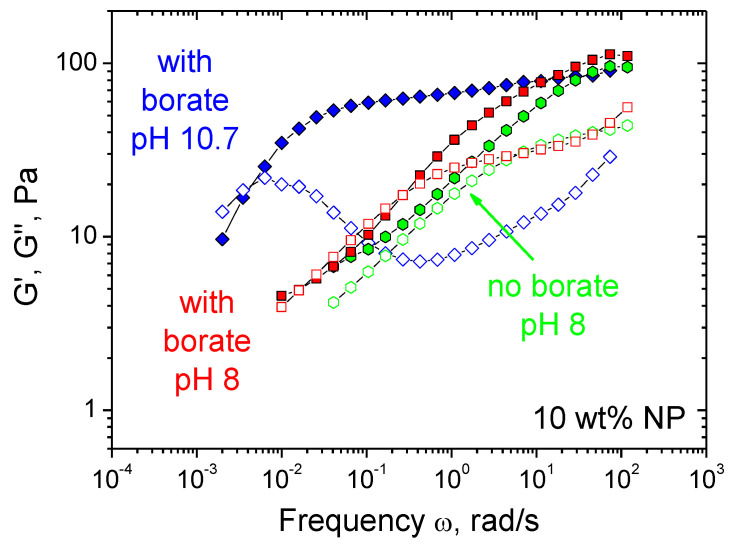
Frequency dependences of storage G′ (filled symbols) and loss G″ (open symbols) moduli for hydrogels containing 1 wt% CMHPG, 10 wt% CoFe_2_O_4_ NPs in the presence of 0.116 wt% borax (molar ratio borate/monomer units = 1) at pH 10.7 (diamonds) or 8 (squares) and in the absence of borax at pH 8 (hexagons). Temperature: 20 °C.

Since borate cross-links are pH-cleavable [34], it is of interest to study the effect of pH on the rheological properties of CMHPG/borate/NP gels. Figure 8 shows the frequency dependencies of storage and loss moduli of the gels at different pH of the solutions: 8 and 10.7. It is seen that at pH 10.7 the frequency range corresponding to predominantly elastic response (G′ > G″) is very broad and there is a wide plateau of the G′(ω) curve. At pH 8, the plateau disappears, and the frequency range corresponding to elastic response diminishes. At this pH, the G′(ω) and G″(ω) curves approach those for the same system in the absence of borate, suggesting the disruption of most of the borate cross-links. This is consistent with the literature data for galactomannan/borax gels, indicating that the cross-links persist only at pH higher than 9.0 [51]. At the same time, even in the absence of borate cross-links, the system keeps viscoelastic behavior, which is due to some entanglements between macromolecules and the remaining cross-links by NPs. Therefore, one can suggest that lowering pH from 10.7 to 8 disrupts most borate cross-links, whereas cross-links by NPs still persist in the system. Such behavior can be quite helpful for the application of these gels as actuators, since it helps to reversibly tune the mechanical properties of the gel, making its matrix softer and more responsive to external triggers like magnetic field.

### 3.4. Effect of Magnetic Field on Mechanical Properties

**Figure 9:**
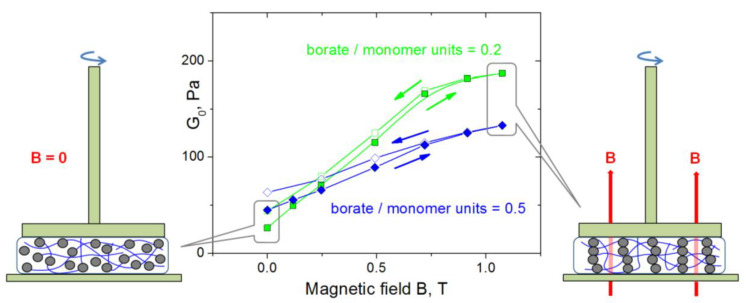
Dependences of G_0_ (storage modulus at ω = 1.7 rad/s) on magnetic field strength for hydrogels containing 1 wt% CMHPG, 10 wt% CoFe_2_O_4_ NPs in the presence of different borax concentrations: 0.023 wt% (corresponding to borate/monomer units = 0.2 − squares) and 0.058 wt% (corresponding to borate/monomer units = 0.5 − diamonds). Temperature: 20 °C.

For the application of CMHPG/borate/NP gels as magnetic-field-triggered actuators, it is important to elucidate the effect of magnetic field on their mechanical properties. In order to investigate this effect, the gels were put into a uniform magnetic field, and their mechanical properties were studied at shear deformations perpendicular to the field. It is seen (Figure 9) that the magnetic field has a strong effect on the mechanical properties: the high-frequency storage modulus G_0_ significantly increases with the rise of the field intensity. These results can be explained as follows. When a uniform magnetic field is applied, NPs acquire magnetization and interact by magnetic forces, moving towards each other and forming chain-like structures, which are oriented along the field force lines (Figure 9). The chains formed by magnetic NPs are oriented perpendicular to shear; therefore, they inhibit the deformation, which is manifested in the increase of the elastic modulus. This effect was previously observed for various magnetic materials, including magnetic elastomers [63], polymer [64] and surfactant hydrogels [65]; however, to the best of our knowledge, it has never been reported for borate cross-linked magnetic gels. Borate cross-links play an important role in high sensitivity of the gels to magnetic field: indeed, borate cross-links, as well as the bonds between polymer chains and NPs, are dynamic and reversible; therefore, NPs are able to move inside the borate-cross-linked polymer matrix to follow the magnetic field.

In the case of a loosely cross-linked polymer matrix (borate/monomer units = 0.2), the effect of magnetic field on the elastic modulus is much stronger than for a tightly cross-linked CMPGH (borate/monomer units = 0.5). This is explained by the fact that at low elasticity of the polymer matrix, NPs can easily move and form well-assembled chains, while for a strongly cross-linked matrix, elastic forces acting on NPs from the polymer network resist their movement and formation of NP chains. In the latter case, the mesh size of the network ξ was estimated from the value of the elastic modulus G_0_ by using the equation [60]

(4) $$G_{0}{=\mathrm{kT}/\xi }^{3}$$

where k is the Boltzmann constant, and T is the absolute temperature. ξ equals to ca. 45 nm, which is comparable to the size of NPs (20 nm). Due to this, the particles are not tightly fixed and are able to move inside the network, but polymer coils serve as obstacles and hamper free movement.

The dependences of G_0_ on the magnetic field show hysteresis: when the magnetic field is reduced, the elastic modulus is higher than for the case of increasing field (Figure 9). This is presumably due to the ferrimagnetic properties of cobalt ferrite NPs: they possess high remanent magnetization (Figure 2B), and when the field is removed, they still interact due to remanent magnetic moments, and chain-like structures are not completely broken. Their partial conservation in the absence of the field may be assisted by the elasticity of the polymer matrix preventing disorganization of NPs. 

Therefore, the mechanical properties of CMHPG/borate/NP magnetic gels can be enhanced by magnetic field, which is due to the formation of NP chains made possible due to the dynamic nature of cross-links in the gels.

### 3.5. Reshaping and Self-Healing

**Figure 10:**
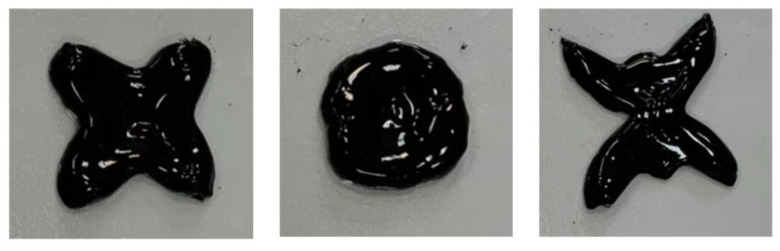
Reshaping of hydrogels containing 1 wt% CMHPG, 10 wt% CoFe_2_O_4_ NPs in the presence of 0.116 wt% borax (molar ratio borate/monomer units = 1) at pH 10.7. Temperature: 20 °C.

A possibility to modify the shape in a controlled manner can play an essential role in the development of functional actuators. Reshaping of CMHPG/borate/NP gels was investigated at room temperature on an example of hydrogel containing 1 wt% CMHPG, 0.116 wt% borax (molar ratio borate/monomer units = 1), and 10 wt% CoFe_2_O_4_ NPs. To modify the shape, the sample was slowly reformed by a spatula. It was observed that the hydrogels can be easily reshaped many times, as shown in Figure 10. The shape deformation occurs instantaneously. It is obviously due to the labile character of cross-links both by borax and by NPs, which under the external force can quickly break and reform in order to keep the new shape. 

**Figure 11:**
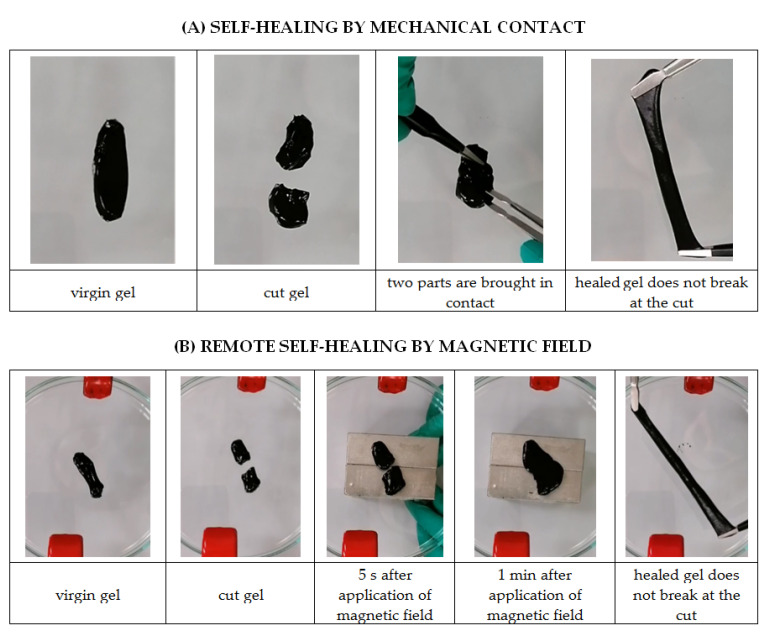
Self-healing of hydrogels containing 1 wt% CMHPG, 10 wt% CoFe_2_O_4_ NPs and 0.116 wt% borax (molar ratio borate/monomer units = 1) at pH 10.7 by bringing two parts in contact (**A**) and remotely in the presence of magnetic field (**B**). Temperature: 20 °C.

Self-healing of CMHPG/borate/NP gels was studied at room temperature by cutting a gel sample in half and placing the two parts in contact for 10 min. During this time, the gel was completely restored. Being stretched, the healed gel did not break at the cut (Figure 11A and Video S1 of Supplementary materials). After 10 min, the gel exhibited 100% healing efficiency with regard to the elongation ratios. Remote self-healing by the magnetic field was also achieved (Figure 11B and Video S2 of Supplementary materials). In this case, two parts of the cut gel were placed in the field of a magnet (0.26 T), and they were brought together remotely by magnetic forces acting on the NPs. Since NPs are bound to the polymer matrix, this resulted in the movement of the gel as a whole. The gel regained conformity in less than 1 min. After 15 min, the self-healed gel was stretched by a factor of 7 without breaking. The observed self-healing properties are due to fast restoration of both types of labile cross-links between polymer chains.

Thus, the CMHPG/borate/NP gels demonstrate the ability to reshape and self-heal, which mimics the healing of muscles and will help to prolong the lifetime of the actuators.

### 3.6. Actuation

**Figure 12:**
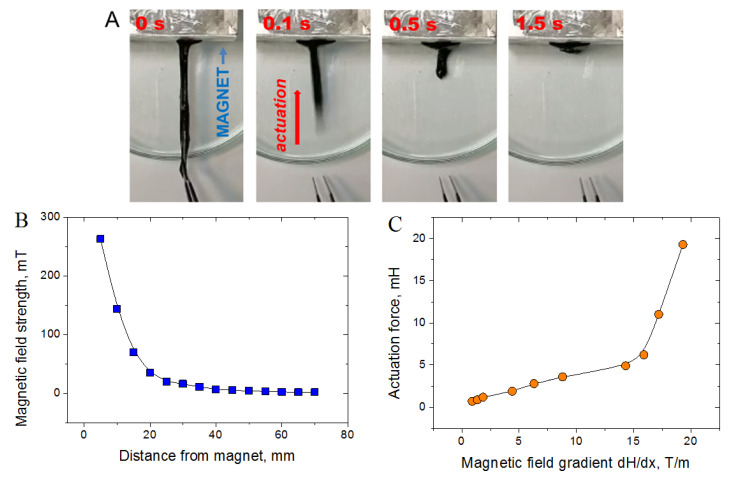
(**A**) Movement of the hydrogel (1 wt% CMHPG, 0.116 wt% borax (molar ratio borate/monomer units = 1), and 10 wt% CoFe_2_O_4_ NPs) in a non-uniform magnetic field of a permanent magnet; (**B**) dependence of the magnetic field strength on the distance from the magnet; (**C**) dependence of the force generated by the gel in the non-uniform magnetic field on the magnetic field gradient (calculated in the center of the gel).

In order to study the actuation capability of CMHPG/borate/NP gels, a strip of the gel (80 mm length, 5×5 mm wide) containing 1 wt% CMHPG, 0.116 wt% borate (molar ratio borate/monomer units = 1), and 10 wt% CoFe_2_O_4_ NPs was prepared. This strip was put in the non-uniform magnetic field of a permanent magnet (Figure 12A), so that one end of the strip was located close to the magnet pole. When the other end of the strip, located far from the pole, was released, the gel contracted very rapidly—in 0.5 s, it decreased its length by a factor of 5, meaning that the gel shows a very fast actuation capability. This is due to the movement of magnetic NPs in the non-uniform magnetic field, which tend to displace along the field gradient, e.g., attract to the magnet pole. Since NPs are bound to the polymer matrix, this causes the movement of the gel as a whole. Large and fast deformations of the gel are possible due to the dynamic nature of borate and NPs cross-links, which reversibly break and adapt to the movement of the material.

To quantify the actuation capability of the gels, the force generated by them in the magnetic field was measured. Both ends of the gel stripe were fixed, and the magnetic field acting on the gel was varied by changing the distance from the magnet (Figure 12B). Another end of the gel stripe was connected to a force meter. From Figure 12C, it is seen that the force generated by the gel rises with the increase of the magnetic field gradient, which is evidently due to stronger magnetic forces acting on NPs. It should be noted that the actuation force appears in rather low magnetic fields (up to 0.26 T) and low field gradients (15–20 T/m). Such fields may be created by small magnets, which makes CMHPG/borax/NP gels promising for use as compact actuators with controllable actuation force. In order to generate the force, direct contact of the gel with the magnet is not necessary, which means that the remote actuation (through some surfaces or non-magnetic materials) may be achieved.

A combination of high deformability and strong and fast response is possible due to the utilization of very small cobalt ferrite NPs, which have high magnetization and at the same time increase the mechanical strength of the gels, and the use of dynamic borate cross-links between polymer molecules.

## 4. Conclusions

Hydrogels with strong and fast response to the magnetic field were prepared from CMHPG polysaccharide dually cross-linked by cobalt ferrite NPs and borate ions, both types of cross-links being labile and easily reformable. It is shown that multifunctional NP cross-links enhance the mechanical properties of the gels. At the same time, the lability of cross-links imparts such useful functional properties as self-healing and reshaping as well as fast response to external stimuli. It is discovered that the mechanical properties of the gels are magnetically tunable: their elastic modulus increases in the external magnetic field, and the strongest increase is observed for the case of the polymer matrix loosely cross-linked by borate. This is explained by the fact that NPs can move inside the polymer matrix cross-linked by labile borate bonds and adapt to the change of the external field. In addition to magnetoresponsive properties provided by NPs, the gels exhibit pH-sensitivity provided by pH-cleavable borate cross-links. Multiresponsive properties and fast reaction to the triggers make the gel prospective for various actuator and sensing applications. Future efforts in this area may be directed at the cross-linking of polymer carboxylic functional groups (for instance, by multivalent metal ions) and obtaining mechanically tough and multiresponsive gels triply cross-linked by metal ions, borate, and NPs.

## Data Availability

The data presented in this study are available on request from the corresponding author.

## Supplementary Materials

The following are available online at https://www.mdpi.com/article/10.3390/nano11051271/s1, Video S1: self-healing of gels by mechanical contact, Video S2: self-healing of gels by magnetic field.

## Abbreviations

CMHPG	carboxymethyl hydroxypropyl guar
HPG	hydroxypropyl guar
NP	nanoparticle
TEM	transmission electron microscopy
HR-TEM	high resolution transmission electron microscopy
